# Commentary: Comparison of the Long-Term Effect of Positioning the Cathode in tDCS in Tinnitus Patients

**DOI:** 10.3389/fnagi.2017.00443

**Published:** 2018-01-11

**Authors:** Yang-soo Yoon, Byungkwan Hwang, Allison Coltisor

**Affiliations:** ^1^Department of Speech, Language, and Hearing Sciences, Texas Tech University Health Sciences Center, Lubbock, TX, United States; ^2^Department of Neurology, Texas Tech University Health Sciences Center, Lubbock, TX, United States

**Keywords:** tinnitus, tDCS, dorsolateral prefrontal cortex, tinnitus functional index, hippocampus

The present study (Rabau et al., 2017) compared the effect of transcranial direct current stimulation (tDCS) on chronic tinnitus between bifrontal group [i.e., Anode and Cathode were placed on the right and left dorsolateral prefrontal cortex (DLPFC), respectively] and shoulder group (i.e., Anode was placed on the right DLPFC, but Cathode on the left upper arm). The dependent measures included Tinnitus Functional Index (TFI) scores as a primary outcome and visual analog scales (VAS) and Hyperacusis questionnaires as a secondary outcome. The study showed no significant difference between two stimulation groups on all outcomes. In this commentary, we address one meritorious approach and a few areas for further consideration and observation of potential significant results.

Many previous tinnitus studies have focused on measuring acute outcomes even though tinnitus is neurologically recursive. There is a need to include appropriate follow-up measures in tinnitus intervention studies. The current study successfully conducted follow-up assessment of the effects of tDCS at the 84-day post-treatment point. Of the studies examining tDCS effects on tinnitus, several with follow-up outcome measures found that there were significant decreases in Tinnitus Handicap Inventory scores between 4 months to 1 year following treatment measures (Henry et al., 2005; Wazen et al., 2011). With one study demonstrating temporary treatment effects only (Chung et al., 2012). Due to the mixed results across studies, it is imperative that researchers establish strict follow-up measures in order to demonstrate long-term benefit for patients following the end of the prescribed treatment period or intervention regimen.

While tDCS is a clinically feasible and implementable intervention for tinnitus, there remains a need for its efficacy to be verified using objective measures prior to being dismissed as inefficacious. Tinnitus is a symptom of physiological damage that causes neurological changes and abnormal neural pathway activation. These changes can objectively be measured by using brain imaging and brain wave techniques (van der Loo et al., 2009; San Juan et al., 2017). By using Functional Near-Infrared Spectroscopy, San Juan et al. (2017) showed “an altered pattern of spontaneous brain connectivity that is differentially regulated following sound stimulation” when compared with controls. To strengthen the protocol administered in the current study, the researchers could partner the VAS with electroencephalogram (EEG) measures as van der Loo et al. (2009) found that subjective perception on the VAS correlated to the resting state EEG in tinnitus patients.

As discussed in the current study (Rabau et al., 2017), it is important to understand the complex neurological pathway activation present in tinnitus patients. Tinnitus activates both cortical (i.e., prefrontal, parietal, and frontal) and subcortical (thalamic and limbic systems) areas along with Heschl's gyrus (Schlee et al., 2009; Vanneste et al., 2010; De Ridder et al., 2011; Eggermont and Roberts, 2012). While the hippocampus, the targeted area in this study, is known to play a role in the perception of prominent affective features of tinnitus (Elgoyhen et al., 2012), the researchers also targeted the secondary pathway responsible (i.e., other components of the limbic system) for the perception of tinnitus, making focused stimulation difficult. The possibility exists that the mediation of the distress network through tDCS may not be as beneficial as mediation provided from the primary neurological response pathway as the distress network activation in tinnitus is secondary (Vanneste et al., 2010; De Ridder et al., 2011). An improved significance level may be achieved through treatment targeting the site of primary auditory lesion (or as close to the site of lesion as possible) in order to both mediate the symptoms in a more efficacious manner and suppress the perpetuation of undesirable cortical activation that commonly occurs in patients with tinnitus.

Based on the distribution of the electric field during tDCS in the current study (Rabau et al., 2017), the bifrontal group was primarily stimulated on the hippocampus, while the stimulation of shoulder group was focused on the temporal lobe, cingulate cortex, and right side of hippocampus. Stimulations were primarily applied to subcortical areas known to be responsible for negative emotional-affective and memory domains in the brain, with the exception of the temporal lobe (de Andrade et al., 2011). It is well-documented that tinnitus is strongly indicated as a factor influencing negative emotions such as anxiety and depression (Eggermont and Roberts, 2014; Neugebauer, 2015). To strengthen future tDCS studies, it is necessary to assess how tDCS stimulation influences negative emotions and memory in tinnitus patients. For example, the effect of the tDCS treatment on tinnitus may be quantified by measuring TFI and assessment of changes in TFI scores may be reflected on the level of anxiety, quantified by relating normative ratings of emotions for a set of anxiety-sensitive images to scores of the TFI (Doehrmann et al., 2013). The association of severity of tinnitus with the level of depression will be quantified by determining correlations between scores of TFI and Beck Depression Inventory. A more interesting question may be how changes in emotion influence the degree to which tDCS affects the severity of tinnitus.

Previous studies have demonstrated that using well-established psychoacoustic measurements such as pitch matching, loudness matching, minimum masking levels, and residual inhibition can help quantify tinnitus treatment outcome and effectiveness (Henry and Meikle, 2000; Newman et al., 2014). Nevertheless, these psychoacoustic measures of the sensory aspects of tinnitus are less commonly used, in part because they are more time consuming and require specific acoustic equipment and protocols (Henry and Meikle, 2000). Assessing changes in the psychoacoustic properties of tinnitus as a result of treatment is an integral part to finding an appropriate and effective treatment for their tinnitus. Clinically, there appear to be significant differences between patients with regard to the degree to which they focus on the sensory aspects using psychoacoustic measures vs. the functional effects of their tinnitus using questionnaires. The fact that measures of those two aspects of tinnitus experience—sensory impairment vs. functional disability and handicap—each provide unique insights into treatment-related changes in tinnitus and reinforce the notion that both approaches are needed for insightful assessment of tinnitus treatment outcomes.

## Author contributions

YY contributed to overall framework of the work and psychoacoutic components of the tinnitus measure, BH contributed to the aspect of the cortical stimulation points, and AC contributed the points of the objective measures in tinnitus treatment.

### Conflict of interest statement

The authors declare that the research was conducted in the absence of any commercial or financial relationships that could be construed as a potential conflict of interest. The handling Editor declared a shared affiliation, though no other collaboration, with one of the authors YY.
